# Determinants of substance use among young people attending primary health centers in India

**DOI:** 10.1017/gmh.2024.13

**Published:** 2024-02-12

**Authors:** U. Venkatesh, P. Aparnavi, K.A. Mogan, R. Durga, Jennifer Pearson, Surekha Kishore, Hari Shanker Joshi, Naveen Sukumaran Nair, B. Nisha, Renu Agrawal, Karavadi Vidusha, C. Vankhuma Chenkual, Bhola Nath, Venkata Rao Epari, Ranjeeta Kumari, Pooja Goyal, Farhad Ahamed, Madhurjya Baruah, R. Anil, Amrut Arun Swami, Bhushan Dattatray Kamble, Gopal Ashish Sharma, Akash Sharma, Om Prakash Bera, Ashoo Grover, Shikhar Kishore Verma, FASAI Study Group

**Affiliations:** 1 Department of Community & Family Medicine, All India Institute of Medical Sciences, Gorakhpur, India; 2 Kovai Medical Center and Hospital Institute of Health Sciences and Research, Coimbatore, India; 3 South Asia Field Epidemiology and Technology Network, Inc., Metro Manila, Philippines; 4 Department of Dentistry, All India Institute of Medical Sciences, Gorakhpur, India; 5 School of Public Health University of Nevada, Reno, NV, USA; 6 All India Institute of Medical Sciences (AIIMS), Gorakhpur, India; 7 Mount Zion Medical College Hospital, Enadimangalam, Kerela; 8 Saveetha Medical College and Hospital, Kuthambakkam, India; 9 Sarojini Naidu Medical College, Agra, India; 10 Rajarajeswari Medical College and Hospital, Bangalore, India; 11 Zoram Medical College, Mizoram, India; 12 All India Institute of Medical Sciences, Raebareli, India; 13 Institute of Medical Sciences and Sum Hospital, Bhubaneswar, India; 14 All India Institute of Medical Sciences, Rishikesh, India; 15 Employment State Insurance Corporation Medical College and Hospital, Faridabad, India; 16 All India Institute of Medical Sciences, Kalyani, India; 17 Lakhimpur Medical College and Hospital, Assam, India; 18 PES Institute of Medical Sciences and Research, Beggilipalle, India; 19 Zydus Medical College and Hospital, Dahod, India; 20 All India Institute of Medical Sciences, Bibinagar, India; 21 Indira Gandhi Medical College, Shimla, India; 22 Department of Medicine, University at Buffalo – Catholic Health System, Buffalo, NY, USA; 23 Global Health Advocacy Incubator, Washington, DC, USA; 24 Indian Council of Medical Research, New Delhi, India; 25 Independent Researcher

**Keywords:** substance use, young people, adolescents, tobacco, WHO-ASSIST

## Abstract

**Background:**

Substance use is a complex condition with multidimensional determinants. The present study aims to find the prevalence and determinants of substance use among young people attending primary healthcare centers in India.

**Methods:**

A multicentric cross-sectional study was conducted across 15 states in India on 1,630 young people (10–24 years) attending primary health centers. The Alcohol, Smoking, and Substance Involvement Screening Test (ASSIST) was used to capture data on substance use. The degree of substance involvement was assessed and multivariate regression analysis was conducted to determine the risk factors of substance use.

**Results:**

The prevalence of substance use was 32.8%, with a median substance initiation age of 18 years. Among the substance users, 75.5% began before completing adolescence. Tobacco (26.4%), alcohol (26.1%) and cannabis (9.5%) were commonly consumed. Sociodemographic determinants included higher age, male gender, urban residence, positive family history, northeastern state residence and lower socioeconomic class. Over 80% of users had moderate or high involvement.

**Conclusions:**

High substance use prevalence among young people in Indian healthcare centers underscores the urgency of targeted intervention. Insights on determinants guide effective prevention strategies for this complex public health issue.

## Graphical abstract

**Figure:**
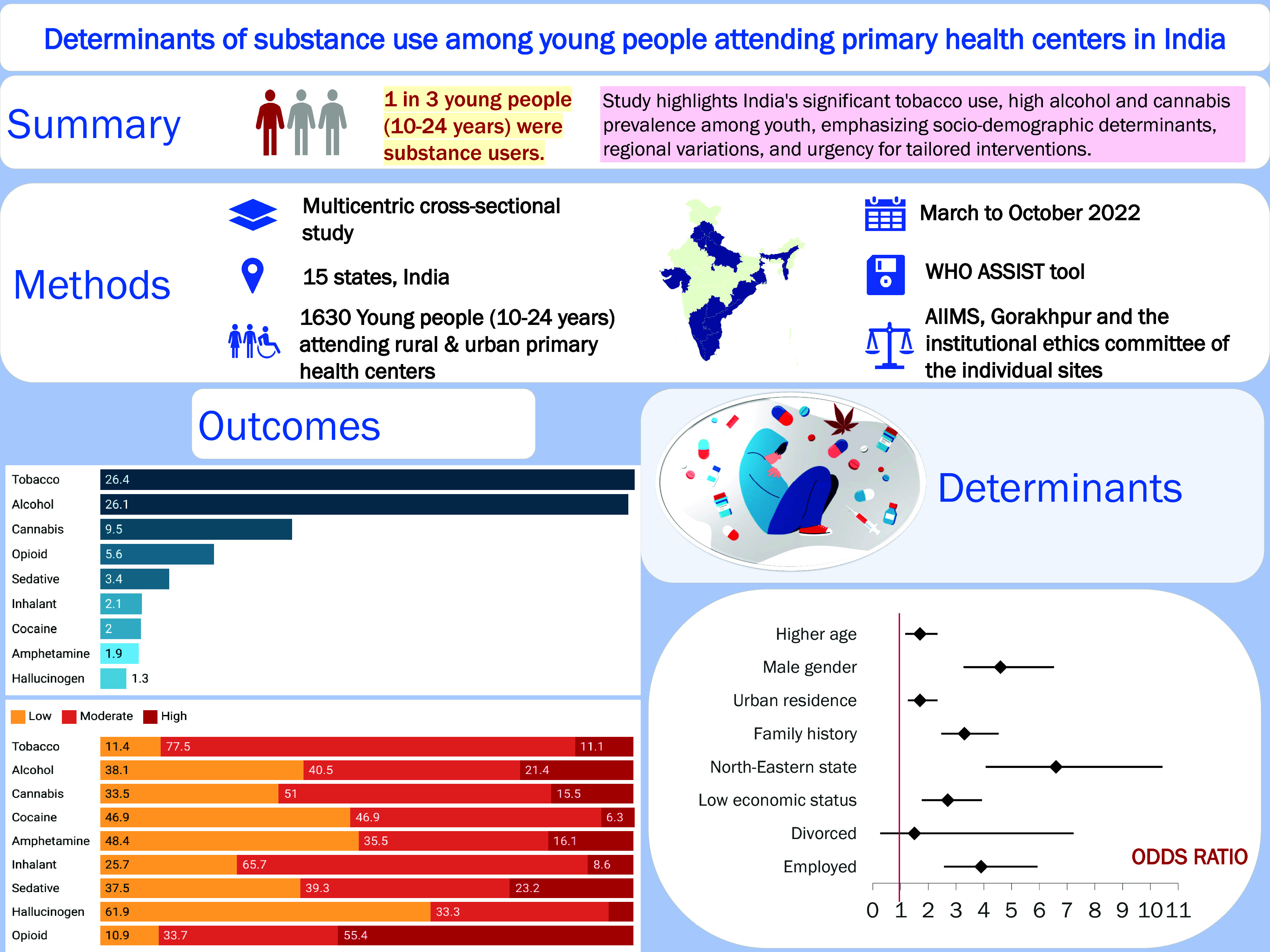

## Impact statement

Our study findings align with the global scenario where tobacco use remains a significant public health concern. India, being the second largest consumer of tobacco globally, faces substantial health risks associated with its use. The study also highlights the high prevalence of alcohol and cannabis use among young individuals. The early initiation of substance use underscores the urgency of targeted interventions during early adolescence. The sociodemographic determinants identified, such as age, male gender, urban residence, family history, and lower socioeconomic class, provide valuable insights for developing targeted prevention and intervention strategies. These determinants mirror findings from previous research, emphasizing the need for multifaceted approaches that consider social, economic and cultural factors influencing substance use. The study’s geographical variation in substance use prevalence, with Mizoram having the highest and Kerala having the lowest, suggests the importance of regional context in understanding and addressing substance use patterns. The findings underscore the need for tailored interventions for regional differences and cultural nuances. The severity of substance involvement, with more than 80% of users falling into the moderate or high involvement categories, signals the urgency for comprehensive and multi-pronged interventions. The study’s use of the WHO ASSIST tool provides a nuanced understanding of substance involvement across various substances, allowing for targeted interventions based on the specific patterns observed. The study’s emphasis on early adolescence as a critical period for intervention aligns with existing evidence that early substance use initiation can lead to dependence, affecting psychosocial behavior, physical health and mental well-being. The call for interventions addressing the accessibility of substances emphasizes the importance of policy measures to restrict easy access, especially considering that friends are reported as the primary source of introduction to substances.

## 1. Background

Substance use includes the misuse of prescription drugs; the use of tobacco, alcohol and illicit drugs (i.e., cocaine, heroin, methamphetamines, inhalants, hallucinogens or ecstasy); and the use of injection drugs (WHO, 2010; McLellan, 2017) Whereas substance abuse is the hazardous use of substances in amounts such that it causes physical or mental harm (WHO, 2023a). In 2017, it was reported that around 5.5% of the global population aged 15–64 had used substances, and about 35 million people were estimated to be affected by substance use disorders. Globally, half a million deaths annually are attributable to substance use and contribute about 1.3% of the disease burden (WHO, 2023b). Tobacco use is the single largest source of preventable deaths worldwide. Though alcohol is the most common substance used worldwide, tobacco is highly prevalent in India. India is the second largest tobacco consumer globally, and around one-fourth of adults in India consume tobacco (Centres for Disease Control and Prevention (CDC), 2016). Among persons aged 13–18 years, the prevalence of ever tobacco users was 18.5% and about half started tobacco before the age of 10 (Government of India (GoI), 2019a). Few other studies showed that a majority of adults initiate substance use in their youth (Sharma and Tyagi, 2016). In current trends, cannabis use among youth has been gradually increasing, and very few studies were conducted to assess the pattern and determinants (Kuepper et al., 2011).

Studying substance use in adolescence is crucial due to heightened vulnerability to initiation during this critical developmental phase. Early substance use can lead to enduring physical, mental and cognitive impacts, elevating the risk of future substance use disorders and associated problems like educational underachievement (Balyakina et al., 2014; Baingana et al., 2015). Behaviors established during this period often persist into adulthood, emphasizing the need for early identification and intervention to prevent harmful habits. Moreover, the profound developmental changes in the adolescent brain, especially in decision-making and impulse control areas, underscore the long-term consequences of substance use. Social dynamics and peer influence significantly contribute to substance initiation among youth, influencing the design of targeted interventions. Substance use among young people not only poses individual risks but also contributes to broader public health issues, necessitating strategies that address societal impacts (Tsering et al., 2010; Mogan et al., 2020). Research in this area informs evidence-based policies and prevention efforts tailored to specific age groups, presenting valuable opportunities for early intervention programs that mitigate the negative consequences of substance use (Boys et al., 2001; Chandler et al., 2009; United States, 2016; Santangelo et al., 2022). Primary health centers (PHCs) serve as the frontline of healthcare and first point of contact with community, making them pivotal in identifying and addressing substance use issues early on. Early detection and intervention at PHCs can prevent the escalation of substance use disorders, reducing the burden on higher levels of healthcare. Moreover, integrating substance use studies into PHCs aligns with a comprehensive approach to healthcare, addressing not only physical health but also mental and behavioral aspects, thus promoting holistic well-being in the community. Furthermore, while national surveys offer insights into the prevalence of individual substance use, they do not conduct a comprehensive examination of all types of substance use within a single study. Additionally, there is a limited exploration of the factors associated with substance use in these surveys. The present study aimed to estimate the prevalence and determinants of substance use among young people attending rural and urban primary health centers in India.

## 2. Methods

### 2.1 Study design, setting and population

**Figure 1.**
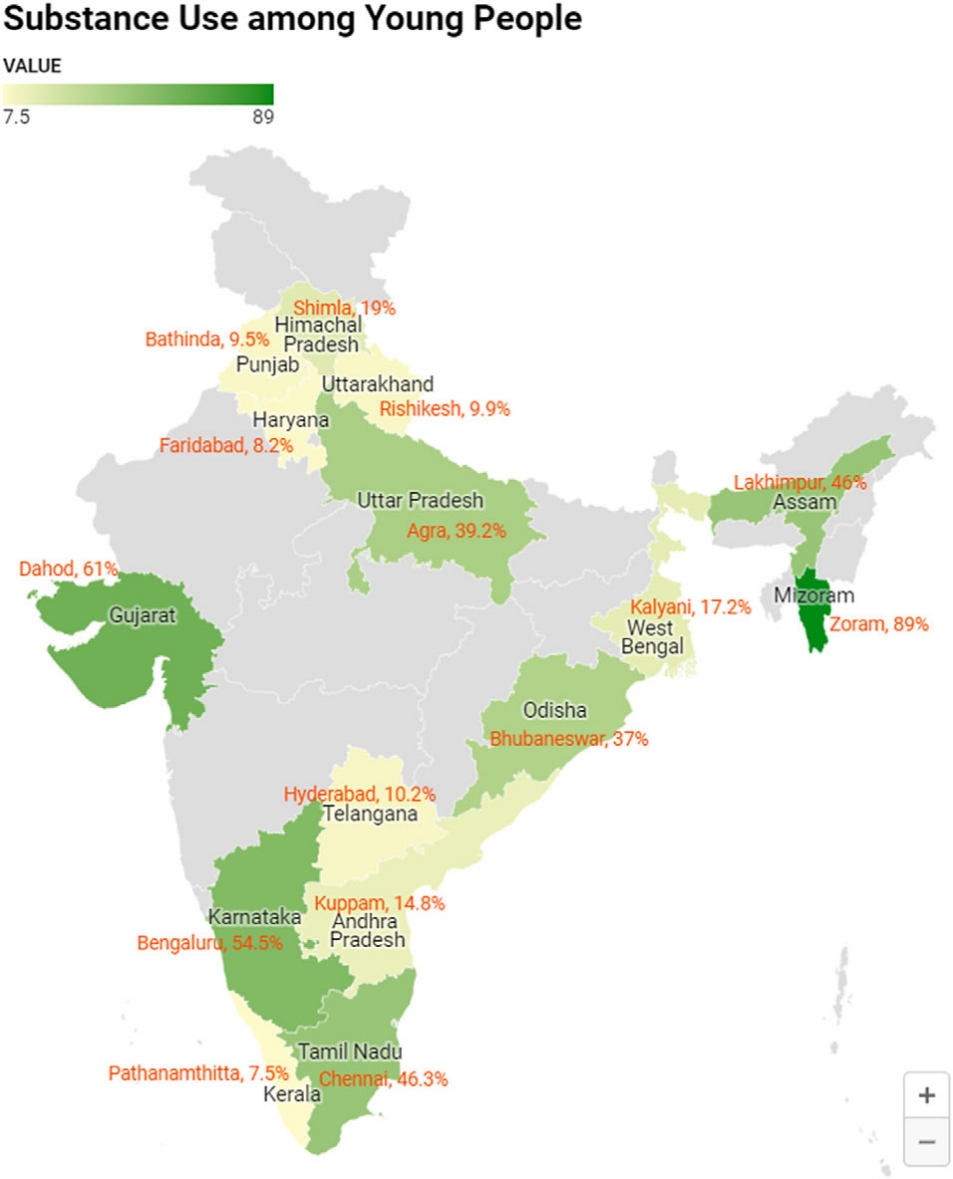
Map of India showing the distribution of substance users (*N* = 1,630). *Note*: Tobacco was consumed by 26.4% (430/1630), followed by alcohol (26.1%, 424/1630) and cannabis (9.5%, 155/1630). More than one substance was consumed by 22.2% (362/1630). Injectable drugs were used by 3.5% (58/1630), which was 11% (58/524) of the substance users. The median substance use score as per the ASSIST tool was the highest for opioids at 29.5 (12–36) and lowest for hallucinating drugs at 3 (0–6). Tobacco and alcohol had median scores of 15 (11–22) and 16 (6–26), respectively. Among opioid users, majority (55.4%, 51/92) were in the high involvement category (Table 1). Substances were introduced to the users majorly by friends (87.2%, 457/524), followed by family members (7.4%, 39/524). Less than 5% (26/524) started using substances by themselves, either through the internet or accidentally at parties.

We conducted a multicenter, cross-sectional study across 15 states in India (one medical college from each state) from March to October 2022 (Figure 1, Supplementary material S2). The participants were young people (10–24 years) attending rural and urban primary health centers of those colleges. Only those in need of emergency management were excluded from the study. The eligible participants were chosen by consecutive sampling.

### 2.2 Sample size and sampling technique

The sample size was calculated using “Epi-info,” a public domain software developed by the Centers for Disease Control (CDC) version 7. Considering the NFHS 5 data for tobacco use (rural) of 42.7%, 95% confidence level and 5% confidence limit, the calculated sample size was 376. Accounting for a design effect of 4.0, based on a study by Emilie et al. (Shea et al., 2009) for multicentric studies, the minimum sample size required was 1,504 from 15 study sites. The study sites were chosen by convenience sampling, representing all five zones and the northeastern (NE) part of India (Government of India (GoI), 2022).

### 2.3 Study tools and data collection

After a review of literature and discussion with subject experts, a predesigned, semi-structured questionnaire was used to capture sociodemographic details and determinants of substance use. The World Health Organization Alcohol, Smoking, and Substance Involvement Screening Test (WHO ASSIST) in clinical setting (v3.1) was used to assess the participants for substance use (World Health Organization, 2010). ASSIST involves screening for tobacco products, alcoholic beverages, cannabis, cocaine, amphetamine-type stimulants, inhalants, sedatives, hallucinogens, opioids and intravenous substance use based on lifetime use. Substance involvement score was calculated individually for each substance and divided into grades 1 (low), 2 (moderate) and 3 (high) based on the use of substances in the past 3 months as per the ASSIST guidelines. The ASSIST tool showed 95% sensitivity and specificity of 79%–93% (Gryczynski et al., 2015). Brahm Govind (BG) Prasad’s socioeconomic scale (SES) was first proposed in 1961 and updated real time based on the Consumer Price Index (CPI). BG Prasad scale with reference CPI values as of January 2022 was used for calculating the socioeconomic class (Bashar, 2022). By norms, the medical colleges in India have rural and urban training at PHCs. All eligible participants attending OPD for their illness were approached and explained about the study. Data was obtained by one-on-one interview administered via a Google form questionnaire.

*Operational definitions*: Substance use was defined as the use of licit or illicit substances other than when medically indicated, such as tobacco, alcohol, cannabis, cocaine, amphetamines, inhalants, sleeping pills, hallucinogens, opioids and intravenous drugs (World Health Organization 2010). The standard WHO definition of young people as an overlap of adolescents (10–19 years) and youth (19–24 years), which included those 10–24 years, was adopted (WHO, 202,3).

### 2.4 Data analysis and statistical methods

Data was checked for completeness and errors and analyzed using Statistical Package for Social Sciences (SPSS) software (version 21.0), acquired by the International Business Machines (IBM), New York, USA. As per the ASSIT tool, the prevalence of substance use was calculated based on self-reported data for lifetime use. The scoring of substance use was based on the past 3-month usage. A person involved in moderate to high use of any one of the substances was considered in the moderate to high involvement category. The prevalence of substance use was calculated and mean scores were calculated for substance involvement scores. Logistic regression was done to quantify the association between substance use and covariates such as age, sex, education, occupation, family history of substance use, socioeconomic status and family type. The association was expressed as odds ratio (OR) with a 95% confidence interval (CI). For multivariate regression in assessing both substance use and severity of involvement, adjustment was done for age, gender, type of family, residence, marital status, family history, category of state, education or employment status and the socioeconomic class (the model fit showed significant omnibus test and nonsignificant Hosmer and Lemeshow test values).

### 2.5 Ethical issues

Ethical clearance was obtained from the institutional ethics committee of both the primary institute (AIIMS, Gorakhpur) and of the individual sites. The WHO ASSIST in a clinical setting (v3.1) was used after obtaining permission from the World Health Organization (WHO) (request ID 390056). Written informed consent from adults and assent from accompanying parents in case of minors was obtained before enrolment into the study. If there was no parent accompanying, the study was explained to the minors and, if willing to participate, they were asked to come along with their parents to the PHC. The privacy of the participants was ensured during data collection. After data collection, a brief intervention was given based on the standard guidelines of ASSIST.

## 3. Results

The study was performed on a sample of 1,630 participants in the age group of 10–24 years from 15 states across India. The mean age of the participants was 19.5 ± 3.5 years. Around one-third (33%, 539/1630) of the participants belonged to the lower SES as per BG Prasad’s classification. Substance use was observed in family members of 44.5% (726/1630). Tobacco was the most common (31.9%, 521/1630) substance used by family members, followed by alcohol (25%, 408/1630).

The overall prevalence of substance use was 32.8% (524/1630) (Figure 1). Females had a lower prevalence (12.2%, 80/656) of substance use. The mean age of initiating substances was 17.2 ± 2.7 years, with no significant difference between males (17.6 years) and females (17.2 years). Among the substance users, 24.6% (129/524) had started use before 16 years of age, and 75.5% (396/524) started before they completed adolescence (<20 years). Christians had a higher prevalence (50.3%, 163/324) of substance use, followed by Hindus (29.4%, 320/1088), people with “Other” religious beliefs (24.4%, 19/78) and Muslims (15.7%, 22/140) (Table 1). Mizoram had the highest prevalence of substance use (89%, 121/136), and Kerala the least (7.5%, 25/332) (Figure 1). In the northeastern states, 75.3% (150/199) were substance users; of them, 68.3% (136/199) were Christians. In other words, among Christians in northeastern states, 88.9% (121/136) were substance users.

**Table 1.** Prevalence of substance use and level of involvement (*N* = 1,630)

Type of substance	Prevalence of use (%)	Use in the past 3 months (%)	Substance involvement score among users		
			Low	Moderate	High
Tobacco	431 (26.4)	382 (23.4)	49 (11.4)	334 (77.5)	48 (11.1)
Alcohol	425 (26.1)	356 (21.8)	162 (38.1)	172 (40.5)	91 (21.4)
Cannabis	155 (9.5)	109 (6.6)	52 (33.5)	79 (51)	24 (15.5)
Cocaine	32 (2)	17 (1.0)	15 (46.9)	15 (46.9)	2 (6.3)
Amphetamine	31 (1.9)	16 (0.9)	15 (48.4)	11 (35.5)	5 (16.1)
Inhalant	35 (2.1)	17 (1.0)	9 (25.7)	23 (65.7)	3 (8.6)
Sedative	56 (3.4)	35 (2.1)	21 (37.5)	22 (39.3)	13 (23.2)
Hallucinogen	21 (1.3)	9 (0.5)	13 (61.9)	7 (33.3)	1 (4.8)
Opioid	92 (5.6)	73 (4.4)	10 (10.9)	31 (33.7)	51 (55.4)
Other drugs*	17 (1.0)	8 (0.4)	9 (52.9)	7 (41.2)	1 (5.9)

* Coollip, Dextromethorphan, Hans.

People in the age group of 20–24 years, males, urban residents, northeastern residence, married, employed, those with a positive family history and falling under lower socioeconomic class had a significantly higher proportion of substance use (Table 2). Among the substance users, 67% had a positive family history of substance use. Though family type and marital status were significant on bivariate analysis, it was not so on adjusting for other variables (Table 2).

**Table 2.** Sociodemographic profile of substance users and non-users (*N* = 1,630)

Sociodemographic	Substance use (row %)	Non-use (row %)	Unadjusted OR (95% CI), *p-*value*^#^*	Adjusted OR (95% CI), *p-*value*^#^*
Age				
10–19 years	114 (16.4)	581 (83.6)	Ref	
20–24 years	410 (43.9)	525 (56.1)	**3.9 (3.1–5), *<0.01***	**1.7 (1.2–2.3), *<0.01***
Gender				
Female	80 (12.2)	576 (87.8)	Ref	
Male	444 (45.6)	530 (54.1)	**6.03 (4.6–7.8), *<0.01***	**4.6 (3.3–6.5), *<0.01***
Family type				
Nuclear	347 (29.8)	817 (70.2)	Ref	
Joint	165 (37.8)	272 (62.2)	**1.4 (1.1–1.7), *0.002***	0.8 (0.5–1.1), *0.2*
Other*	12 (41.4)	17 (58.6)	1.6 (0.7–3.5), *0.2*	1 (0.3–2.7), 0.4
Residence				
Rural	261 (26.6)	719 (73.4)	Ref	
Urban	263 (40.5)	387 (59.5)	**1.8 (1.5–2.3), *<0.01***	**1.7 (1.3–2.3), *<0.01***
Marital status				
Never married	391 (27.8)	1,014 (72.2)	Ref	
Currently married	111 (55.5)	89 (44.5)	**3.2 (2.3–4.4), *<0.01***	1.9 (0.4–8.7), *0.1*
Divorced/widowed	22 (88)	3 (12)	**19 (5.6–63.8), *<0.01***	1.5 (0.3–7.2), 0.3
Family history				
No	173 (19.1)	731 (80.9)	Ref	
Yes	351 (48.3)	375 (51.7)	**3.9 (3.1–4.9), *<0.01***	**3.3 (2.5–4.5), *<0.01***
State groups				
Other states	374 (26.1)	1,057 (73.9)	Ref	
Northeastern	150 (75.4)	49 (24.6)	**8.6 (6.1–12.1), *<0.01***	**6.6 (4.1–10.4), *<0.01***
Education/employment status				
Currently studying	160 (16)	841 (84)	Ref	
Employed	250 (60.1)	166 (39.9)	**7.9 (6.1–10.2), *<0.01***	**3.9 (2.6–5.9), *<0.01***
Unemployed and not studying	114 (53.5)	99 (46.5)	**6.1 (4.4–8.3), *<0.01***	1 (0.7–1.6), *0.14*
Socioeconomic status				
I (upper)	14 (23.7)	45 (76.3)	Ref	
II (upper middle)	70 (28)	180 (72)	1.2 (0.6–2.4),0.5	1.0(0.4–2.2),0.9
III (middle)	116 (29.3)	280 (70.7)	1.3 (0.7–2.5),0.4	1.1(0.4–2.3),0.8
IV (lower middle)	105 (27.2)	281 (72.8)	1.2 (0.6–2.2),0.6	0.8(0.4–1.9),0.8
V (lower)	219 (40.6)	320 (59.4)	**2.1 (1.1–4.1), 0.01**	**2.7 (1.8–3.9), *<0.01***

# Logistic regression.

* Broken families/have moved out of family/do not have family.

Bold: Statistically significant.

Nearly one-third reported that tobacco and alcohol products were easily accessible. One to three percent reported that substances, including cocaine, amphetamine, inhalants, sedatives, hallucinating drugs and opioids, were available within their premises (Table 3).

**Table 3.** Accessibility to individual substances (*N* = 1,630)

Type of substance	In a nearby store	On an online site	On their premises	Through a third person	From friends/social circle	Do not know how to access/not willing to disclose
Tobacco	534	9	512	2	61	641
	(32.8)	(0.6)	(31.4)	(0.1)	(3.7)	(39.3)
Alcohol	658	42	150	57	100	893
	(40.4)	(2.6)	(9.2)	(3.5)	(6.1)	(54.7)
Cannabis	262	45	111	35	70	1,324
	(16.1)	(2.8)	(6.8)	(2.2)	(4.3)	(81.2)
Cocaine	43	19	46	21	16	1,540
	(2.6)	(1.2)	(2.8)	(1.3)	(1)	(94.4)
Amphetamine	16	23	25	16	11	1,602
	(1)	(1.4)	(1.5)	(1)	(0.7)	(98.2)
Inhalant	30	22	17	10	13	1,555
	(1.8)	(1.3)	(1)	(0.6)	(0.8)	(95.3)
Sedative	39	23	16	13	11	1,582
	(2.4)	(1.4)	(1)	(0.8)	(0.7)	(97.0)
Hallucinogen	23	23	18	20	12	1,541
	(1.4)	(1.4)	(1.1)	(1.2)	(0.7)	(94.5)
Opioid	17	22	50	21	15	1,588
	(1)	(1.3)	(3)	(1.3)	(0.9)	(97.4)
Other drugs	41	22	26	12	11	1,599
	(2.5)	(1.3)	(1.6)	(0.7)	(0.7)	(98.1)

*Note*: Multiple choices were applicable, hence not mutually exclusive.

Family history of substance use, place of residence and type of family did not statistically affect the severity of tobacco score. Among the substance users, 437 (83.4%) were in the moderate or high involvement category for at least one of the substances, and 16.6% were in the low involvement category for all the substances they were using. Higher age, male gender and educational/employment status were significantly associated with the severity of substance involvement after adjusting for other variables (Table 4).

**Table 4.** Overall severity score for substance across the sociodemographic profile of participants (*N* = 524)

Sociodemographic	Moderate to high involvement in any of the substances	Low involvement in all substances (%)	Unadjusted OR (95% CI)	Adjusted OR (95% CI)
Age				
10–19 years	88 (77.2)	26 (22.8)	Ref	
20–24 years	349 (85.1)	61 (14.9)	**1.7(1.0–2.8), *0.04***	**1.1(1.6–2), *0.08***
Gender				
Female	46 (57.5)	34 (42.5)	Ref	
Male	391 (88.1)	53 (11.9)	**5.4(3.2–9.2), *<0.01***	**4.9(2.7–8.8), *<0.01***
Family type				
Joint	140 (84.8)	25 (15.2)	Ref	
Nuclear/single parent	297 (82.7)	62 (17.3)	1.2(0.7–2.0), *0.4*	0.9(0.5–1.7), *0.9*
Residence				
Rural	213 (81.6)	48 (18.4)	Ref	
Urban	224 (85.2)	39 (14.8)	1.3(0.8–2.1), *0.27*	1.5(0.9–2.6), *0.09*
Marital status				
Never married	318 (81.3)	73 (18.7)	Ref	
Ever married	119 (89.5)	14 (10.5)	0.6(0.3–1.16), *0.1*	1.2(0.6–2.5), *0.5*
Family history				
No	138 (79.8)	35 (20.2)	Ref	
Yes	299 (85.2)	52 (14.8)	1.5(0.9–2.3), *0.1*	1.2(0.7–2.2), *0.5*
State groups				
Other states	303 (81)	71 (19)	Ref	
Northeastern	134 (89.3)	16 (10.7)	**1.9(1.1–3.5), *<0.01***	1.8(0.9–3.5), *0.1*
Education/employment status				
Currently studying	113 (70.6)	47 (29.4)	Ref	
Employed	224 (89.6)	26 (10.4)	**3.5(2.1–6.1), *<0.01***	**2.4(1.2–4.6)*, 0.01***
Unemployed and not currently studying	100 (87.7)	14 (12.3)	**2.9(1.5–5.7), *<0.01***	**2.7(1.3–5.7), *0.07***
Socioeconomic status				
I (upper)	14 (3.2)	0 (0)	7.7(0.4–132.7), *0.2*	1.2(0.3–4.6), 0.8
II (upper middle)	60 (85.7)	10 (14.3)	1.5(0.8–3.4), *0.2*	0.7(0.3–1.7), *0.5*
III (middle)	100 (86.2)	16 (13.8)	1.6(0.8–3.1), *0.2*	0.7(0.3–1.7), *0.6*
IV (lower middle)	90 (85.7)	15 (14.3)	1.5(0.8–3.0), *0.1*	0.8(0.3–1.9), *0.8*
V (lower)	173 (79)	46 (21)	Ref	

Bold: Statistically significant.

Among the substance users, 210 (40.1%) were willing to take help for quitting, and another 212 (19.5%) reported that they might consider taking help.

## 4. Discussion

The present study reports that the prevalence of substance use among people in the age group of 10–24 years is 32.8%. Corroborating with the current findings, the study by Baba et al. (2013) on college students showed a prevalence of about 31.3%. The prevalence rates in our study exceeded those found in studies on school children in Turkey (21.4%) (Pumariega et al., 2014) and Brazil (27.3%) (Malta et al., 2011) as well as a study on male adolescents in Aligarh (13.3% vs 45.6% males in the current study) (Ahmad et al., 2009). While the inclusion of higher age groups in our study may explain the variance, higher prevalence rates (58.7% and 53.8%) were observed in two Indian studies (Juyal et al., 2008; Hembram et al., 2015).

The current finding that more than a quarter (26.4%) of young people use substances was in accordance with the global GATS 2 reported prevalence (28.6%) among individuals aged 15 years and above (Centres for Disease Control and Prevention (CDC), 2016). Notably, GATS reports the initiation age for tobacco use at 20.9 years. Consequently, a meaningful comparison of the prevalence rates between the current study and GATS can be undertaken taking into account the age range and reported age of tobacco initiation in the respective studies. The 2019 GYTS also reported a slightly higher prevalence of tobacco users (8.5%) compared to the current prevalence (4.6%) among the same age group of 13–15 years (Government of India (GoI), 2019b). In a study on pre-university students (Bhojani et al., 2009), the prevalence of tobacco use was 15.7%. A study in one of the northeastern states among school children reported that 46% have ever used tobacco (Ningombam et al., 2011). This might be because of the high prevalence of substance use in the northeastern states. The current study reported a significantly higher prevalence (75.4%) of substance use in northeastern states, which was in accordance with other reports from northeastern India 54% (Ningombam et al., 2011) and 70.8% (Saikia and Debbarma, 2020). The higher prevalence in these states may be attributed to their porous borders, making legal enforcements more challenging, and more recreational tourism. The current study showed that tobacco was the most common substance used, followed by alcohol. Similar reports were evidenced in few other studies also although the source of samples for each of these studies was different (Ningombam et al., 2011; Hembram et al., 2015; Rahman and Tripathi, 2016; Mogan et al., 2020). Christians were the highest proportion of substance users, and of the total Christians, 74.2% are residing in northeastern states. A study among the students of Manipur (Ningombam et al., 2011) reported that substance use was significantly lower among children of Hindu/Jain religion. Hence, the prevalence among Christians was high probably due to the high representation of Christians in northeastern states, which had more substance users.

The prevalence of substance use was much lower in the current study among those actively enrolled in school or college, and this conclusion was validated by two other studies (Rani et al., 2003; Mogan et al., 2020) that also reported that tobacco use was inversely related to education. This can also be explained by the enforcement of the Cigarettes and Other Tobacco Products Act (COTPA) and other rules related to substance usage in educational institutes (Government of India (GoI), 2003). The current study reported that the prevalence was significantly high among those with a positive family history of substance use, and a similar report was provided by a substance use study in Manipur (Ningombam et al., 2011). In the current study, friends played a major role in the introduction of substances. The role of peers in adolescent behaviors, including substance use, is well established (Kobus, 2003; Ningombam et al., 2011; Stritzel, 2022). The current study reports a higher prevalence of cannabis use (9.5%) than a national survey done by National Drug Dependence Treatment Centre (NDDTC) of AIIMS Delhi (2.8%) (Government of India (GoI), 2019b). The NDDTC conducted a survey encompassing a population sample of over 4.5 lakh across all states, while the present study focused on only half of the states than included in the NDDTC survey. Delhi and Haryana, being one of the major cannabis consumers (Government of India (GoI), 2019b), reported only 4.3% (Mogan et al., 2020) and 8.5% (Qadri et al., 2010), respectively. In alignment with the higher overall substance use, though cannabis use was also higher (35.1%) in northeastern states, it was lower than that found by the study in Manipur (14%) (Ningombam et al., 2011). The difference might be because of the study population, as the latter (Ningombam et al., 2011) exclusively studied school population. Though Kashmir also has hurdles in legal enforcement (Baba et al., 2013), it reported lower cannabis use (4.4%).

On regression analysis, the current study reported that higher age group, male gender, place of residence, family history of substance use, geographical area, education or employment status and lower socioeconomic status were all found to be significantly associated with substance use. A study on similar population of young people attending rural health centers showed an association between substance use and male sex, lower socioeconomic level and family history of substance use (Mogan et al., 2020). Male gender, higher age group and urban predominance of substance use were also reported in another study (Qadri et al., 2010). Though too few studies (Qadri et al., 2010) showed that substance use was more common in nuclear families, our study reported that it was significantly higher in joint families and broken families. The higher prevalence in the male gender and higher age group may be due to the increased social exposure in this group. The current relation of substance use with a positive family history was also supported by other literature (Singh and Gupta, 2006; Saxena et al., 2010; Pramod and Narayan, 2019). This reinforces the fact that family has an important role in determining the lifestyle/habits of children.

Tobacco use was significantly higher among those over 19 years of age, men, those who had never been married, people of the low socioeconomic group and those who were not living with family currently (Supplementary file 3). Literature shows that an increased incidence of cigarette use was seen among people in the lower socioeconomic group (Rani et al., 2003; Mogan et al., 2020). This might be due to a lack of awareness about the ill health effects of tobacco among the lower socioeconomic group. The study could have been further expanded to assess the reason for starting substance use, associated injuries, adverse events on the road or sexual and mental behavior. Both the proportion of users and the degree of involvement were high in male gender. This might be due to social norms and stigmatization of female substance users in the Indian society. However, underreporting among females might also be a possibility. More number of people in the 20–24 years group age had higher substance involvement scores. This might indicate that the chances of addiction are higher as age increases and that interventions should be planned for younger people.

While the present study provides valuable insights into the determinants of substance use among young people, there are limitations inherent in the study design. The present study was done on those attending healthcare centers and also used a convenient sampling technique to select the study sites. Hence, this may have introduced selection bias, limiting the generalizability of the findings. The study relied on self-reported data collection, which could have introduced bias affecting the accuracy of responses to certain questions related to substance use. Due to the cross-sectional design, the study was unable to establish the temporality and specificity of associations observed between determinants and substance use, thus limiting its ability to establish causality. Further research with robust study designs and more diverse samples is warranted to better understand the complex dynamics of substance use in this region.

## 5. Conclusion

Our findings show that one-third of young people attending primary health centers engaged in substance use, with two-thirds of them initiating substance use before completing adolescence. The role of family and peers is crucial in the initiation of substance use, as two-thirds of substance users had at least one parent using substance. Notably 90% of substance users reported being introduced to these substances by friends. Higher age, male gender, urban residence, a positive family history, residing in a northeastern state and belonging to a lower socioeconomic class were determinants of substance use. To effectively combat substance use, the study recommends a multifaceted approach. Early intervention programs targeting adolescents should instill awareness and coping mechanisms against peer pressure. Comprehensive educational campaigns emphasizing the risks of substance use, particularly regarding tobacco and alcohol, are crucial. Stricter access control measures, especially in high-use regions, are recommended, including restricting underage access. Community-based initiatives promoting a healthy environment and positive peer influences should be established. Parental education programs to recognize signs of substance use and enhance communication are vital. Increased accessibility to counseling services, research and monitoring, employment opportunities, evidence-based policies and collaborative efforts between stakeholders will further contribute to mitigating substance use.

## Supporting information

Venkatesh et al. supplementary material 1 Venkatesh et al. supplementary material

Venkatesh et al. supplementary material 2 Venkatesh et al. supplementary material

Venkatesh et al. supplementary material 3 Venkatesh et al. supplementary material

## Data Availability

Data and material supporting the results reported in the article can be obtained on reasonable request.

## Supplementary material

The supplementary material for this article can be found at http://doi.org/10.1017/gmh.2024.13.
