# Supplementary material for: Determinants of substance use among young people attending primary health centers in India
Source: Glob Ment Health (Camb). 2024 Feb 12;11:e23. doi: 10.1017/gmh.2024.13 (PMC10988150; doi:10.1017/gmh.2024.13)
Supplement: Venkatesh et al. supplementary material 1 — Venkatesh et al. supplementary material [file S205442512400013Xsup001.docx]

**Supplementary file 1:** Study tool.

**Determinants of substance use among young people attending primary health centres in India**

**Form number: Study state: Mobile number:**

**Name: Age** (in completed years) **: Gender : Religion: Place of Residence: Rural / Urban**

**Marital status :** Currently Married /Currently Unmarried / Divorced or Widowed

**Educational status:**

1. Not literate (can not read and write with understanding in any language)
2. Just literate (can read and write in any one language but has not received formal education at least up to 5th class)
3. Completed primary school (5th class)
4. Completed middle school (8th class)
5. Completed 10th std
6. Completed higher secondary School
7. Graduate / Diploma
8. Post graduate

**Occupational status:**

1. Currently attending School /College
2. Unskilled labor
3. Semi skilled labor
4. Skilled Labor
5. Semi professional
6. Professional
7. Unemployed and not currently studying

**Total family income / month: Total family members:**

**What type of family are you living in?** Nuclear / Joint / Broken families

**Are you currently living with your family?** Yes / No

Are any of your **family members currently** (used in the last 30 days) using **any substance?** (tobacco, Alcohol, Cannabis, Cocaine, Amphetamine, Inhalants, sedatives, Hallucinogens, Opioids, etc.): Yes / No

If Yes,

What is the type of substance that the family members use: Tobacco / Alcohol / Others (specify)_______

Who are the family member using substance? Father / Mother / Sibling / Others(specify)____________

How accessible are these drugs to you? (Tick all that apply)

| **Substance** | **Available in a store such as a convenience store, supermarket, discount store, gas station** | **Available easily on online /**  **ecommerce site** | **Need to buy through third person due to stigma / legal ineligibility** | **Can be used from my peer / social circle** | **Don't know how to access** |
| --- | --- | --- | --- | --- | --- |
| Tobacco |  |  |  |  |  |
| Alcohol |  |  |  |  |  |
| Cannabis |  |  |  |  |  |
| Cocaine |  |  |  |  |  |
| Amphetamine |  |  |  |  |  |
| Inhalants |  |  |  |  |  |
| Sedatives |  |  |  |  |  |
| Hallucinogens |  |  |  |  |  |
| Opioids |  |  |  |  |  |
| Others (specify)______ |  |  |  |  |  |

If Yes to any of the above proceed below. If No to all the substance mentioned above, tis will be the end of survey. Thank you

Have you ever used any of the following substance? (NON- MEDICAL USE ONLY)

(Tick the appropriate cell)

| **Substance** | **Yes** | **No** |
| --- | --- | --- |
| Tobacco |  |  |
| Alcohol |  |  |
| Cannabis |  |  |
| Cocaine |  |  |
| Amphetamine |  |  |
| Inhalants |  |  |
| Sedatives |  |  |
| Hallucinogens |  |  |
| Opioids |  |  |
| Others (specify)______ |  |  |

At what age did you start using drugs (In completed years)

How were you introduced to substance? Friends / Partner / Family / Internet / Other(specify)

In the past three months, how often have you used the substances you mentioned (FIRST DRUG, SECOND DRUG, ETC)?

| **Substance** | **Never** | **Once or Twice** | **Monthly** | **Weekly** | **Daily or Almost** |
| --- | --- | --- | --- | --- | --- |
| Tobacco products (cigarettes, chewing tobacco, cigars, etc.) |  |  |  |  |  |
| Alcoholic beverages (beer, wine, spirits, etc.) |  |  |  |  |  |
| Cannabis (marijuana, pot, grass, hash, etc.) |  |  |  |  |  |
| Cocaine (coke, crack, etc.) |  |  |  |  |  |
| Amphetamine type stimulants (speed, meth, ecstasy, etc.) |  |  |  |  |  |
| Inhalants (nitrous, glue, petrol, paint thinner, etc.) |  |  |  |  |  |
| Sedatives or Sleeping Pills (Diazepam, Alprazolam, Flunitrazepam, Midazolam etc.) |  |  |  |  |  |
| Hallucinogens (LSD, acid, mushrooms, trips, Ketamine, etc.) |  |  |  |  |  |
| Opioids (heroin, morphine, methadone, Buprenorphine, codeine, etc.) |  |  |  |  |  |
| Others |  |  |  |  |  |

During the past three months, how often have you had a strong desire or urge to use(FIRST DRUG, SECOND DRUG, ETC)?

| **Substance** | **Never** | **Once or Twice** | **Monthly** | **Weekly** | **Daily or Almost** |
| --- | --- | --- | --- | --- | --- |
| Tobacco products (cigarettes, chewing tobacco, cigars, etc.) |  |  |  |  |  |
| Alcoholic beverages (beer, wine, spirits, etc.) |  |  |  |  |  |
| Cannabis (marijuana, pot, grass, hash, etc.) |  |  |  |  |  |
| Cocaine (coke, crack, etc.) |  |  |  |  |  |
| Amphetamine type stimulants (speed, meth, ecstasy, etc.) |  |  |  |  |  |
| Inhalants (nitrous, glue, petrol, paint thinner, etc.) |  |  |  |  |  |
| Sedatives or Sleeping Pills (Diazepam, Alprazolam, Flunitrazepam, Midazolam etc.) |  |  |  |  |  |
| Hallucinogens (LSD, acid, mushrooms, trips, Ketamine, etc.) |  |  |  |  |  |
| Opioids (heroin, morphine, methadone, Buprenorphine, codeine, etc.) |  |  |  |  |  |
| Others |  |  |  |  |  |

During the past three months, how often has your use of (FIRST DRUG, SECOND DRUG, ETC)led to health, social, legal or financial problems?

| **Substance** | **Never** | **Once or Twice** | **Monthly** | **Weekly** | **Daily or Almost** |
| --- | --- | --- | --- | --- | --- |
| Tobacco products (cigarettes, chewing tobacco, cigars, etc.) |  |  |  |  |  |
| Alcoholic beverages (beer, wine, spirits, etc.) |  |  |  |  |  |
| Cannabis (marijuana, pot, grass, hash, etc.) |  |  |  |  |  |
| Cocaine (coke, crack, etc.) |  |  |  |  |  |
| Amphetamine type stimulants (speed, meth, ecstasy, etc.) |  |  |  |  |  |
| Inhalants (nitrous, glue, petrol, paint thinner, etc.) |  |  |  |  |  |
| Sedatives or Sleeping Pills (Diazepam, Alprazolam, Flunitrazepam, Midazolam etc.) |  |  |  |  |  |
| Hallucinogens (LSD, acid, mushrooms, trips, Ketamine, etc.) |  |  |  |  |  |
| Opioids (heroin, morphine, methadone, Buprenorphine, codeine, etc.) |  |  |  |  |  |
| Others |  |  |  |  |  |

During the past three months, how often have you failed to do what was normally expected of you because of your use of (FIRST DRUG, SECOND DRUG, ETC)?

| **Substance** | **Never** | **Once or Twice** | **Monthly** | **Weekly** | **Daily or Almost** |
| --- | --- | --- | --- | --- | --- |
| Tobacco products (cigarettes, chewing tobacco, cigars, etc.) |  |  |  |  |  |
| Alcoholic beverages (beer, wine, spirits, etc.) |  |  |  |  |  |
| Cannabis (marijuana, pot, grass, hash, etc.) |  |  |  |  |  |
| Cocaine (coke, crack, etc.) |  |  |  |  |  |
| Amphetamine type stimulants (speed, meth, ecstasy, etc.) |  |  |  |  |  |
| Inhalants (nitrous, glue, petrol, paint thinner, etc.) |  |  |  |  |  |
| Sedatives or Sleeping Pills (Diazepam, Alprazolam, Flunitrazepam, Midazolam etc.) |  |  |  |  |  |
| Hallucinogens (LSD, acid, mushrooms, trips, Ketamine, etc.) |  |  |  |  |  |
| Opioids (heroin, morphine, methadone, Buprenorphine, codeine, etc.) |  |  |  |  |  |
| Others |  |  |  |  |  |

Has a friend or relative or anyone else ever expressed concern about your use of (FIRST DRUG, SECOND DRUG, ETC.)?

| **Substance** | **Yes, in the past 3 months** | **Yes, but not in the past 3 months** |
| --- | --- | --- |
| Tobacco products (cigarettes, chewing tobacco, cigars, etc.) |  |  |
| Alcoholic beverages (beer, wine, spirits, etc.) |  |  |
| Cannabis (marijuana, pot, grass, hash, etc.) |  |  |
| Cocaine (coke, crack, etc.) |  |  |
| Amphetamine type stimulants (speed, meth, ecstasy, etc.) |  |  |
| Inhalants (nitrous, glue, petrol, paint thinner, etc.) |  |  |
| Sedatives or Sleeping Pills (Diazepam, Alprazolam, Flunitrazepam, Midazolam etc.) |  |  |
| Hallucinogens (LSD, acid, mushrooms, trips, Ketamine, etc.) |  |  |
| Opioids (heroin, morphine, methadone, Buprenorphine, codeine, etc.) |  |  |
| Others |  |  |

Have you ever tired to cut down on using(FIRST DRUG, SECOND DRUG, ETC) but failed?

| **Substance** | **No, Never** | **Yes, in past 3 months** | **Yes, but not in past 3 months** |
| --- | --- | --- | --- |
| Tobacco products (cigarettes, chewing tobacco, cigars, etc.) |  |  |  |
| Alcoholic beverages (beer, wine, spirits, etc.) |  |  |  |
| Cannabis (marijuana, pot, grass, hash, etc.) |  |  |  |
| Cocaine (coke, crack, etc.) |  |  |  |
| Amphetamine type stimulants (speed, meth, ecstasy, etc.) |  |  |  |
| Inhalants (nitrous, glue, petrol, paint thinner, etc..) |  |  |  |
| Sedatives or Sleeping Pills (Diazepam, Alprazolam, Flunitrazepam, Midazolam etc.) |  |  |  |
| Hallucinogens (LSD, acid, mushrooms, trips, Ketamine, etc.) |  |  |  |
| Opioids (heroin, morphine, methadone, Buprenorphine, codeine, etc.) |  |  |  |
| Others |  |  |  |

Are you currently willing to take help for stopping use? Yes / No /Maybe

Have you ever used any drug by injection?(NON-MEDICAL USE ONLY)

No, Never / Yes, in the past 3 months / Yes, but not in the past 3 months

If clients have injected drugs in the last 3 months,

PATTERN OF INJECTING

1. 4 days per month, on average, over the last 3 months or less
2. More than 4 days per month, on average, over the last 3 months
