# Supplementary material for: Determinants of substance use among young people attending primary health centers in India
Source: Glob Ment Health (Camb). 2024 Feb 12;11:e23. doi: 10.1017/gmh.2024.13 (PMC10988150; doi:10.1017/gmh.2024.13)
Supplement: Venkatesh et al. supplementary material 2 — Venkatesh et al. supplementary material [file S205442512400013Xsup002.docx]

**Supplementary file 2:** Sociodemographic details of the participants.(N=1630)

| **State** | **District** | **10-19 years** | **20-24 years** | **Male** | **Female** | **Total** |
| --- | --- | --- | --- | --- | --- | --- |
| **Zone South** |  |  |  |  |  |  |
| Tamil Nadu | Chennai* | 57 | 133 | 120 | 70 | 190 |
| Andhra Pradesh | Kuppam | 23 | 38 | 48 | 13 | 61 |
| Kerala | Pathanamthitta | 119 | 213 | 245 | 87 | 332 |
| Karnataka | Bengaluru* | 32 | 80 | 70 | 42 | 112 |
| Telangana | Hyderabad* | 31 | 28 | 36 | 23 | 59 |
| **Zone West** |  |  |  |  |  |  |
| Gujarat | Dahod | 10 | 90 | 93 | 7 | 100 |
| **Zone East** |  |  |  |  |  |  |
| Odisha | Bhubaneshwar | 49 | 32 | 79 | 2 | 81 |
| West Bengal | Kalyani | 40 | 24 | 44 | 20 | 64 |
| **Zone Central** |  |  |  |  |  |  |
| Uttar Pradesh | Agra | 51 | 74 | 79 | 46 | 125 |
| Uttarakhand | Rishikesh | 55 | 26 | 47 | 34 | 81 |
| **Zone North** |  |  |  |  |  |  |
| Himachal Pradesh | Shimla | 40 | 18 | 35 | 23 | 58 |
| Punjab | Bathinda | 80 | 15 | 58 | 37 | 95 |
| Haryana | Faridabad* | 57 | 16 | 28 | 45 | 73 |
| **North-east** |  |  |  |  |  |  |
| Mizoram | Zoram | 7 | 129 | 117 | 19 | 136 |
| Assam | Lakhimpur | 44 | 19 | 33 | 30 | 63 |

*Metropolitan cities(26.6%)
