# Supplementary material for: Determinants of substance use among young people attending primary health centers in India
Source: Glob Ment Health (Camb). 2024 Feb 12;11:e23. doi: 10.1017/gmh.2024.13 (PMC10988150; doi:10.1017/gmh.2024.13)
Supplement: Venkatesh et al. supplementary material 3 — Venkatesh et al. supplementary material [file S205442512400013Xsup003.docx]

**Supplementary file 3**: Socio-demographic profile of Tobacco users in low and moderate to high risk of substance involvement. (n=431)

| **Socio-demographic** | **Low risk** | **Moderate risk** | **High risk** | **p-value*** |
| --- | --- | --- | --- | --- |
| **Age** |  |  |  |  |
| 10 – 19 years | 16 (18) | 68 (76.4) | 5 (5.6) | **0.02** |
| 20 – 24 years | 33 (9.6) | 266 (77.8) | 43 (12.6) |  |
| **Gender** |  |  |  |  |
| Female | 19 (35.8) | 31 (58.5) | 3 (5.7) | **< 0.01** |
| Male | 30 (7.9) | 303 (80.2) | 45 (11.9) |  |
| **Religion** |  |  |  |  |
| Hindu | 42 (16.4) | 203 (79.3) | 11 (4.3) | **<0.01** |
| Muslim | 2 (9.5) | 18 (85.7) | 1 (4.8) |  |
| Christian | 4 (2.8) | 103 (72) | 36 (25.2) |  |
| Others | 1 (9.1) | 10 (90.9) | 0 (0) |  |
| **Family type** |  |  |  |  |
| Nuclear | 31 (11.3) | 210 (76.4) | 34 (12.4) | 0.63 |
| Joint | 18 (12.3) | 115 (78.8) | 13 (8.9) |  |
| Other | 0 (0) | 9 (90) | 1 (10) |  |
| **Currently with family/not** | |  |  |  |
| Yes | 39 (11.9) | 259 (79.2) | 29 (8.9) | **0.02** |
| No | 10 (9.6) | 75 (72.1) | 19 (18.3) |  |
| **Residence** |  |  |  |  |
| Rural | 30 (13.8) | 161 (74.2) | 26 (12) | 0.2 |
| Urban | 19 (8.9) | 173 (80.8) | 22 (10.3) |  |
| **Marital status** |  |  |  |  |
| Currently Married | 12 (11.7) | 79 (76.7) | 12 (11.7) |  |
| Never Married | 37 (12.1) | 243 (79.4) | 26 (8.5) | **<0.01** |
| Divorced / Widowed | 0 (0) | 12 (54.5) | 10 (45.5) |  |
| **Family history of substance use** | |  |  |  |
| Yes | 33 (10.7) | 238 (77.3) | 37 (12) | 0.5 |
| No | 16 (13) | 96 (78) | 11 (8.9) |  |
| **State groups** |  |  |  |  |
| Other states | 33 (11.7) | 236 (84) | 12 (4.3) | **<0.01** |
| North Eastern state | 16 (10.7) | 98 (65.3) | 36 (24) |  |
| **Education / Employment status** | |  |  |  |
| Currently studying | 26 (21.7) | 90 (75) | 4 (3.3) | **<0.01** |
| Employed | 16 (7.5) | 175 (81.8) | 23 (10.7) |  |
| Unemployed and not currently studying | 7 (7.2) | 69 (71.1) | 21 (21.6) |  |
| **Socio-Economic Status** | |  |  |  |
| I (Upper) | 0 (0) | 9 (90) | 1 (10) | **0.04** |
| II (Upper Middle) | 8 (13.1) | 52 (85.2) | 1 (1.6) |  |
| III (Middle) | 13 (12.3) | 85 (80.2) | 8 (7.5) |  |
| IV (Lower Middle) | 9 (10) | 72 (80) | 9 (10) |  |
| V (Lower) | 19 (11.6) | 116 (70.7) | 29 (17.7) |  |

*Chi-square test
