# Supplementary material for: Determinants of substance use among young people attending primary health centers in India
Source: Glob Ment Health (Camb). 2024 Feb 12;11:e23. doi: 10.1017/gmh.2024.13 (PMC10988150; doi:10.1017/gmh.2024.13)
Supplement: Peer review history

# Editor report: Recommendation: Determinants of substance use among young people attending primary health centers in India — R0/PR2

**DOI:** 10.1017/gmh.2024.13.pr2

**Affiliations:** Pravara Institute of Medical Sciences, India

No accompanying comment.

# Editor report: Decision: Determinants of substance use among young people attending primary health centers in India — R0/PR3

**DOI:** 10.1017/gmh.2024.13.pr3

**Affiliations:** London School of Hygiene & Tropical Medicine, United Kingdom of Great Britain and Northern Ireland

No accompanying comment.

# Editor report: Recommendation: Determinants of substance use among young people attending primary health centers in India — R1/PR5

**DOI:** 10.1017/gmh.2024.13.pr5

**Affiliations:** Pravara Institute of Medical Sciences, India

No accompanying comment.

# Editor report: Decision: Determinants of substance use among young people attending primary health centers in India — R1/PR6

**DOI:** 10.1017/gmh.2024.13.pr6

**Affiliations:** London School of Hygiene & Tropical Medicine, United Kingdom of Great Britain and Northern Ireland

No accompanying comment.
